# A water-forming NADH oxidase regulates metabolism in anaerobic fermentation

**DOI:** 10.1186/s13068-016-0517-y

**Published:** 2016-05-11

**Authors:** Xin-Chi Shi, Ya-Nan Zou, Yong Chen, Cheng Zheng, Bing-Bing Li, Jia-Hui Xu, Xiao-Ning Shen, Han-Jie Ying

**Affiliations:** State Key Laboratory of Materials–Oriented Chemical Engineering, College of Biotechnology and Pharmaceutical Engineering, Nanjing Tech University, No. 30, Puzhu South Road, Nanjing, 210009 People’s Republic of China

**Keywords:** Alternative elector acceptor, Anaerobic fermentation, *Clostridium acetobutylicum*, NADH oxidase, *Saccharomyces cerevisiae*

## Abstract

**Background:**

Water-forming NADH oxidase can oxidize cytosolic NADH to NAD^+^, thus relieving cytosolic NADH accumulation in *Saccharomyces cerevisiae*. Previous studies of the enzyme were conducted under aerobic conditions, as O_2_ is the recognized electron acceptor of the enzyme. In order to extend its use in industrial production and to study its effect on anaerobes, the effects of overexpression of this oxidase in *S. cerevisiae* BY4741 and *Clostridium acetobutylicum* 428 (Cac-428) under anaerobic conditions were evaluated.

**Results:**

Glucose was exhausted in the NADH oxidase-overexpressing *S. cerevisiae* strain (Sce-NOX) culture after 26 h, while 43.51 ± 2.18 g/L residual glucose was left in the control strain (Sce-CON) culture at this time point. After 30 h of fermentation, the concentration of ethanol produced by Sce-NOX reached 36.28 ± 1.81 g/L, an increase of 56.38 % as compared to Sce-CON (23.20 ± 1.16 g/L), while the byproduct glycerol was remarkably decreased in the culture of Sce-NOX. In the case of the *C. acetobutylicum* strain (Cac-NOX) overexpressing NADH oxidase, glucose consumption, cell growth rate, and the production of acetone–butanol–ethanol (ABE) all decreased, while the concentrations of acetic acid and butyric acid increased as compared to the control strain (Cac-CON). During fermentation of Cac-CON and Cac-NOX in 100-mL screw-capped bottles, the concentrations of ABE increased with increasing headspace. Additionally, several alternative electron acceptors in *C. acetobutylicum* fermentation were tested. Nitroblue tetrazolium and 2,6-dichloroindophenol were lethiferous to both Cac-CON and Cac-NOX. Methylene blue could relieve the effect caused by the overexpression of the NADH oxidase on the metabolic network of *C. acetobutylicum* strains, while cytochrome c aggravated the effect.

**Conclusions:**

The water-forming NADH oxidase could regulate the metabolism of both the *S. cerevisiae* and the *C. acetobutylicum* strains in anaerobic conditions. Thus, the recombinant *S. cerevisiae* strain might be useful in industrial production. Besides the recognized electron acceptor O_2_, methylene blue and/or the structural analogs may be the alternative elector acceptor of the NADH oxidase in anaerobic conditions.

**Electronic supplementary material:**

The online version of this article (doi:10.1186/s13068-016-0517-y) contains supplementary material, which is available to authorized users.

## Background

In *Saccharomyces cerevisiae*, overflow metabolism begins when the glucose uptake rate exceeds a threshold, and results in the formation of ethanol and glycerol. Glycerol is generated to reoxidize surplus cytosolic NADH that can accumulate during glycolysis because of the rapid consumption of glucose. Cytosolic NADH is reoxidized by two cytosolic NADH dehydrogenases, but when glycolytic NADH generation surpasses the rate at which these dehydrogenases can act, *S. cerevisiae* activates the glycerol synthesis pathway as another outlet for NADH consumption [1].

Recently, the water-forming NADH oxidase-encoding *noxE* gene from *Lactococcus lactis* has attracted substantive attention as it can relieve cytosolic NADH accumulation in *S. cerevisiae*, which does not possess this gene [2, 3]. The use of the heterologous enzyme likely invokes an unbiased response instead of affecting a specific metabolic reaction, which will have localized network effects around the altered reaction [4]. Previous studies showed that overexpression of the water-forming NADH oxidase could increase the consumption of glucose and decrease the accumulation of glycerol in aerobic fermentation [1, 2]. All studies of this enzyme have been conducted under aerobic conditions, because O_2_ is recognized as the optimal electron acceptor of the enzyme. However, aerobic fermentation is unfit for industrial production as the air sparging will remove a large amount of ethanol. Consequently, this study aimed to investigate whether the oxidase works under anaerobic conditions.

Previous studies on *Clostridium acetobutylicum* and *Clostridium aminovalericum*, both obligatory anaerobes, showed that these two strains could grow normally under microoxic (sparged with 3 % O_2_/97 % N_2_ mixed carrier gas) conditions [5, 6]. *Clostridium aminovalericum* has a *noxA* gene, which was strongly upregulated when the growth conditions changed to microoxic, indicating that NoxA is involved in oxygen metabolism. In *C. acetobutylicum*, which has no ortholog of *C.aminovalericum noxA*, Northern blot analysis identified multiple O_2_-responsive genes that were quickly expressed or upregulated when 5 % O_2_ was present.

Instead of oxygen, the water-forming NADH oxidase may have alternative electron acceptors in anaerobic conditions. Although no study has focused on the alternative electron acceptor of the water-forming NADH oxidase to date, there are a few reports on the H_2_O_2_-forming NADH oxidase [7, 8]. Park et al. reported that the H_2_O_2_-forming NADH oxidase purified from the extreme thermophile *Thermus thermophilus* is able to catalyze electron transfer from NADH to various other electron acceptors (methylene blue, cytochrome c, *p*-nitroblue tetrazolium, 2,6-dichloroindophenol, and potassium ferricyanide).

Based on the previous findings, a *S. cerevisiae* strain (Sce-NOX) overexpressing a heterologous water-forming NADH oxidase was constructed. Batch culture growth of the control strain (Sce-CON) and Sce-NOX was compared. To study the role of the NADH oxidase in anaerobic bacteria, the enzyme was overexpressed in *C. acetobutylicum*, a strictly anaerobic gram-positive bacterium [9]. Batch culture growth of the control strain (Cac-CON) and the strain overexpressing NADH oxidase (Cac-NOX) were compared under different oxygen supply conditions. To the best of our knowledge, this is the first study to assess the role of the NADH oxidase in anaerobic condition. Our results showed that overexpression of the NADH oxidase could regulate the metabolism of both the *S. cerevisiae* and the *C. acetobutylicum* strains in anaerobic condition, which can be generalized to other strains.

## Methods

### Construction of the strains

The strains and plasmids used in this study are listed in Table 1. For the *S. cerevisiae* strain, the *noxE* gene from *L. lactis* (GenBank Accession No. AM406671) was PCR-amplified with primers Sce-Nox-F, 5′-*CTTGTGGGCCCA*GGATCCATGAAAATCGTAGTTATCG-3′, and Sce-Nox-R, 5′-*ACAGGAATTCACCAT*GGATCCTTATTTGGCATTCAAAGCTG-3′. Both primers have a *Bam*HI site (underlined), and the homologous arms of the plasmid are indicated in italics in the primer sequences. PCR products were gel-purified and inserted into the *Bam*HI site of plasmid pYX212 by using the ClonExpress™ One Step Cloning Kit (Vazyme Biotech Co., Ltd, Nanjing, China), resulting in pYX212-NOX. The plasmid was transformed into the host strain, BY4741, using G418 (400 μg/mL) to select a stably transfected clone, designated Sce-NOX (Table 1). As a control, Sce-CON, the host strain transfected with empty plasmid was used.

**Table 1:** List of plasmids and strains used in this study

Plasmid/strain	Genotype	Source
Plasmid		
pYX212	2 μ, TPI promoter, AMP^R^	A gift from Pro. Yingjin Yuan (Tianjin University, Tianjin, China)
pYX212-NOX	pYX212 with *noxE* from *L. lactis*	This study
pSY8		Stored in our laboratory
pSY8-NOX	pSY8 with *noxE* from *L. lactis*	This study
Strain		
*S. cerevisiae* BY4741	*MATa;ura3;his3;leu2;met15*	A gift from Pro. Yingjin Yuan (Tianjin University, Tianjin, China)
Sce-CON	BY4741/pYX212	This study
Sce-NOX	BY4741/pYX212-NOX	This study
*C. acetobutylicum* 428		Stored in our laboratory
Cac-CON	*C. acetobutylicum* 428/pSY8	This study
Cac-NOX	*C. acetobutylicum* 428/pSY8-NOX	This study

For the *C. acetobutylicum* strain, the *noxE* gene (AM406671) was PCR-amplified with primers Cac-Nox-F, 5′-*CTGCAGGTCGAC*GGATCCATGAAAATCGTAGTTATC-3′, and Cac-Nox-R, 5′-*TATAGAATTCCCGG*GGATCCTTATTTGGCATTCAAAGCTG-3′. Both primers have a *Bam*HI site (underlined) and the homologous arms of the plasmid are indicated in italics in the primer sequences. Gel-purified PCR products were inserted into the *Bam*HI site of pSY8 by using the ClonExpress™ One Step Cloning Kit, resulting in pSY8-NOX. The plasmid was transformed into *C. acetobutylicum* 428 (CGMCC No. 5234) using thiamphenicol (15 μg/mL) for clone selection. The strains containing the overexpression and empty plasmid were designated Cac-NOX and Cac-CON, respectively (Table 1).

### Media and growth conditions

The yeast strains (Sce-CON, Sce-NOX) were maintained on conventional yeast extract peptone dextrose (YPD) agar plates as described previously [10, 11].

The aerobic seed cultures for cultivation were grown at 30 °C in 500 mL Erlenmeyer flasks containing 100 mL of complex medium A (initial pH 5.2) containing 20 g/L glucose, 10 g/L tryptone (Oxoid), 5 g/L yeast extract (Oxoid), and 9 g/L NaCl [12] in a rotary shaker at 200 rpm.

The anaerobic fermentations for Sce-CON and Sce-NOX were conducted in 100-/250-mL screw-capped bottles with two exhaust pipes, each of which had a filter membrane. The fermentation medium contained 90 g/L glucose, 10 g/L tryptone (Oxoid), 5 g/L yeast extract (Oxoid), and 9 g/L NaCl. The culture condition was 32 °C at 150 rpm. Nitrogen gas was used to flush the medium after inoculation and sampling to ensure anaerobic conditions.

For Cac-CON and Cac-NOX strains, the modified P2 medium for seed culture and the P2 medium for fermentation have been described previously [13]. Artificial dyes were added to the fermentation medium as indicated. In different oxygen supply fermentation conditions, the conditions were as follows: 500-/100-mL Erlenmeyer flask sealed with eight-layer gauze; 500-/100-mL Erlenmeyer flask sealed with eight-layer gauze and one piece of kraft paper; 250-/100-mL Erlenmeyer flask sealed with eight-layer gauze; 250-/100-mL Erlenmeyer flask sealed with eight-layer gauze and one piece of kraft paper; 100-/50-mL screw-capped bottle. All five fermentation cultures were incubated on a rotary shaker at 100 rpm, at 37 °C and stewing in an incubator, respectively.

### Metabolite analyses

The cell density of *S. cerevisiae* was measured at 600 nm using a BioMate™ 3 spectrophotometer (Thermo Scientific, Waltham, MA, USA). Five milliliters of culture was centrifuged at 4000×*g* for 10 min. The supernatants were used to determine the concentrations of glucose and metabolites.

The glucose and glycerol concentrations were measured by high-performance liquid chromatography (Agilent 1100 series; Hewlett–Packard, Palo Alto, CA, USA) with a refractive index detector, using a Benson BP-100 Pb^++^ column (300 × 7.8 mm; Benson Polymeric Inc, Sparks, NV, USA). Ultrapure water was used as the mobile phase at a flow rate of 0.4 mL/min and the column temperature was set at 80 °C.

The solvents (acetone, butanol, and ethanol) and acids (butyric acid and acetic acid) were analyzed using a gas chromatography system (7890A GC-System, Agilent Technologies, Palo Alto, CA, USA) equipped with a flame ionization detector (FID) and a 30-m capillary column (Equity 1™; 30 m × 0.32 mm, 1.0 μm film thickness; Supelco Co, Bellefonate, PA, USA) [13].

### Enzyme activity

Total NADH oxidation activity was assayed spectrophotometrically following the method of Vemuri et al. [1]. A unit of activity was defined as the quantity that catalyzed the oxidation of 1 μmol of NADH per minute. Protein was quantified using the Bradford method using BSA as a standard.

### Quantification of intracellular NAD(P)H/NAD(P)

Intracellular concentrations of NAD(P)H were determined using the enzyme cycling method of Liu et al. [13] with modifications. Generally, two 1 mL samples were taken and cells were collected and dissolved in 0.5 mL of 0.1 M NaOH (to assay NAD(P)H) and 0.5 mL of 0.1 M HCl (to assay NAD(P)), respectively. The cell lysate was heated at 50 °C for 10 min, cooled to 0 °C, and centrifuged at 10,000×*g* for 10 min. The supernatant was used for follow-up measurement.

A mixture of 100 μL Tris–HCl (1 M, pH 7.8), 100 μL 4.2 mM MTT, 150 μL 16.6 mM PES, and 100 μL ethanol for the determination of NAD(H) or 100 μL 60 mM glucose 6-phosphate for the determination of NADP(H) was sequentially added to a test tube and kept at 37 °C for 5 min in the dark. ddH_2_O and a moderate amount of supernatant (75 μL in total) were added to 96-well plates. The plates were preheated at 37 °C for 5 min in a Multi-mode Detection Platform (SpectraMax Paradigm; Molecular Devices, CA, USA). Ten microliters of alcohol dehydrogenase (1.5 units/μL, for NAD(H)) or glucose 6-phosphate dehydrogenase (70 units/mL, for NADP(H)) was added to the mixture, and 46 μL of the mixture was added to the 96-well plates to start the reaction. The absorbance at 570 nm was determined. The production for NADH was measured over 10 min at 2 min intervals, and the production for NADPH, NADP, and NAD was measured over 30 min at 5-min intervals.

### Quantitative reverse transcription (qRT)-PCR analysis

RNA was isolated from cells as described previously [14]. Reverse transcription was performed using the AMV First Strand cDNA Synthesis Kit (Sangon Biotech, Shanghai, China) according to the manufacturer’s instructions. Primer Express software was used for primer design. The analyzed genes and primers used in the analysis are listed in Table 2. qRT-PCR assays were performed with the SYBR Green PCR Master Mix (Applied Biosystems, Foster City, CA, USA) on a StepOnePlus Real-Time PCR System according to the manufacturer’s instructions. Three technical replicates were included for each sample. Gene transcript levels were determined according to the 2^−ΔΔCt^ method, using the *ACT1* gene (for *S. cerevisiae*) [15] and a housekeeping gene-CA_C0279 (for *C. acetobutylicum*) [16] as reference genes for normalizing the gene expression levels. To verify qRT-PCR data, standard deviation values were calculated using Microsoft Excel (Microsoft Corporation, Redmond, WA, USA), and used to evaluate the repeatability and the effectively of these data.

**Table 2:** genes and primers for quantitative real-time PCR

Gene ID	Gene name	Primer sequences
YFL039C	*ACT1* (reference gene)	F: TGGATTCCGGTGATGGTGTT R: TGGCGTGAGGTAGAGAGAAACC
L196579	*noxE*	F: TCAAAAATGGCGCAATCAAG R: CCGCGTAAACATCTGGATCA
YMR169C	*ALD3*	F: TGGCGGCTCAGTATTGGAA R: CGCATTCTAGTGTGATATCCTTAAGG
YPL061W	*ALD6*	F: GACAAAGTCAACGGTAGAACAATCA R: GGCTCTAAGGTGGTGAAGTTCATG
YOL086C	*ADH1*	F: GAAGGTGCCGGTGTCGTT R: ACCGATCTTCCAGCCCTTAAC
YDL022W	*GPD1*	F: TCAATTTTTGCCCCGTATCTG R: GATAGCTCTGACGTGTGAATCAACA
YHL032C	*GUT1*	F: GCCCCAGCTCGTGAAACA R: GGGCTTTCCGCTGGTTTT
YJR009C	*TDH2*	F: TCCAAGAAAGAGACCCAGCTAACT R: GGAGTCAATGGCGATGTCAA
YDR343C	*HXT6*	F: CGCTGCTATTGCAGAGCAAAC R: CGAGTGGGAGGCTGAGTCA
CA_C0279	Housekeeping gene	F: AGAAGTGGGAGCACCTGTAAAAA R: CGGTTCAATCTTTCCTTCAACTTT
L196579	*noxE*	F: TCAAAAATGGCGCAATCAAG R: CCGCGTAAACATCTGGATCA
CA_C1742	*Pta*	F: GAAATTCAGACCGGATCTTGCT R: GCCGCTACTTCACTATCTATTGCA
CA_C1743	*AskA*	F: CAATGGATATAAGTGCAGAAGGTTCTA R: CTTTGGTATCCCTTGCAATCATTA
CA_P0163	*CtfA*	F: CAACCCAGATACTGGCAAAAAAC R: TGCACGTATTCTTTCCACTAGAGTTC
CA_P0165	*Adc*	F: ACGCTATGGCGCCACTTAAT R: TGCAAGAATGTGAGAGCTAGAAACA
CA_C3075	*Buk*	F: GGCGGACTCTTAAAGCCAATAGTA R: GCATGCTGACCTTGAACTCCTA
CA_C3076	*Ptb*	F: CAACACTTGATGCAGCAATGC R: GCTAAAGGTCCGTCAACTACACAA
CA_P0035	*adhE*	F: AGGGAGCAAGCGGAGATTTAT R: TGCCGCATCCAAGAGTAAATG
CA_C0028	*hydA*	F: GGAAAATGCGGAGTCTGTATGG R: TGGCAACACAAGCAGCTCTAA

## Results and discussion

### Anaerobic fermentation by Sce-CON and Sce-NOX

**Fig. 1:**
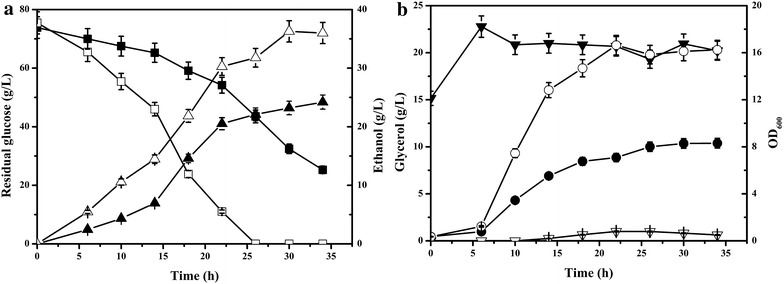
Time course of fermentation by the control strain Sce-CON and the NADH oxidase-overexpressing strain Sce-NOX. **a** *Filled triangle* *closed triangle*, ethanol; *Filled square* *open square*, residual glucose; **b** *Closed circle* *open circle*, OD_600_; *Black down-pointing triangle* *white down pointing triangle*, glycerol. *Solid symbols*, Sce-CON; *open symbols*, Sce-NOX

Batch culture growth of the control strain Sce-CON and the Sce-NOX strain overexpressing NADH oxidase in anaerobic condition was compared (Fig. 1). The glucose consumption rate and cell growth rate of Sce-NOX were higher than those of Sce-CON. The glucose was exhausted at 26 h of Sce-NOX culture, while 43.51 ± 2.18 g/L residual glucose remained in the Sce-CON culture at this time point. In addition, after 30 h of fermentation, the concentration of ethanol produced by Sce-NOX reached a peak value of 36.28 ± 1.81 g/L, which was 56.38 % higher than that of Sce-CON at the same time point (23.20 ± 1.16 g/L). The production of the byproduct glycerol by Sce-NOX was remarkably lower, which was in accordance with previous reports of increased assimilation of NADH in the cytosol by NADH oxidase, leading to reduced glycerol production [1, 3]. The glycerol concentration of Sce-NOX remained below 1 g/L in both seed culture and anaerobic fermentation. For Sce-CON, a large amount of glycerol was produced in both culture processes; the accumulation of glycerol in the fermentation process was approximately five times higher than that in Sce-NOX culture.

**Fig. 2:**
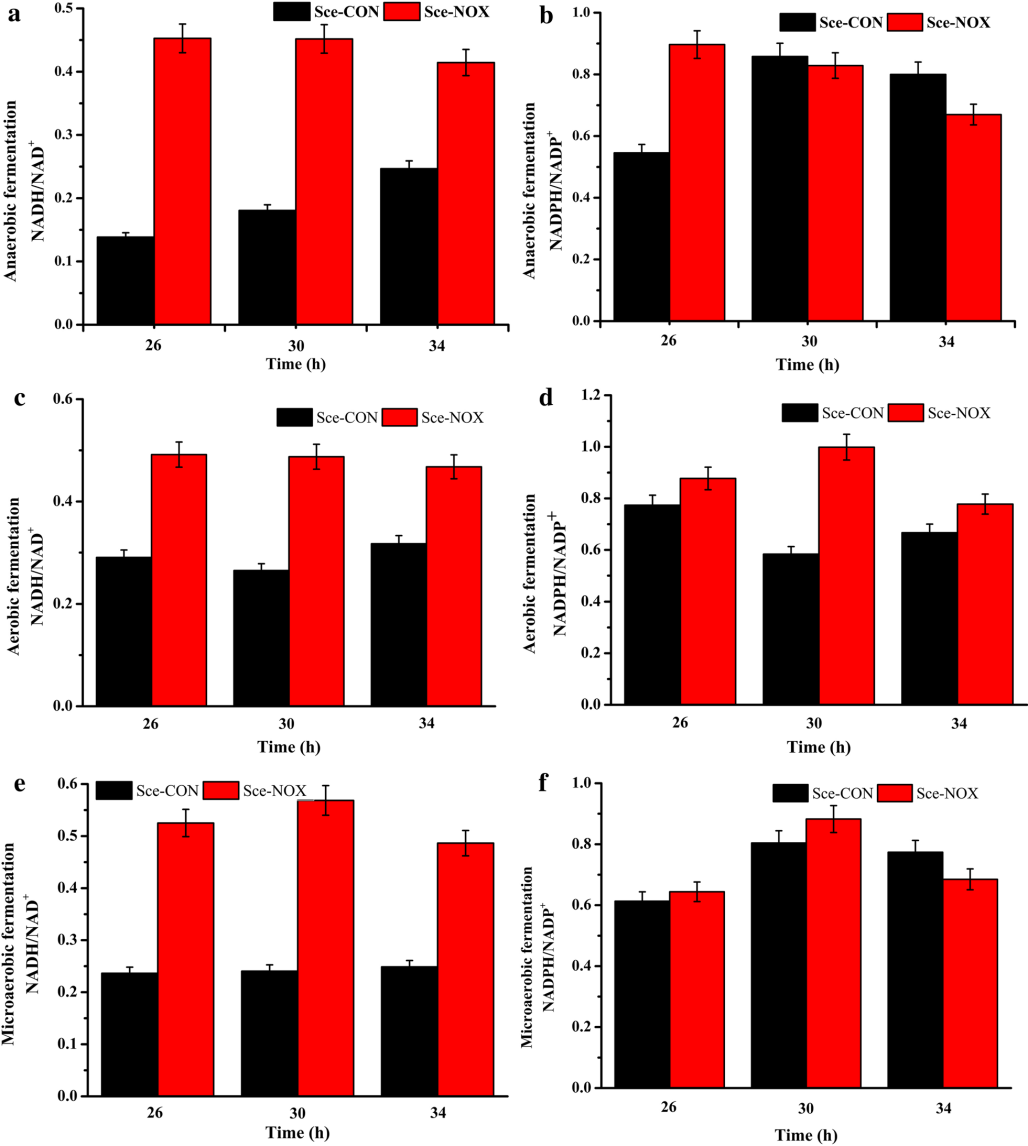
NADH/NAD^+^ and NADPH/NADP^+^ ratios of Sce-CON and Sce-NOX. In the batch fermentation in different oxygen supply models, the concentrations of the intracellular cofactors of Sce-CON and Sce-NOX at 26, 30, and 34 h were measured. **a** The NADH/NAD^+^ ratio in anaerobic fermentation; **b** The NADPH/NADP^+^ ratio in anaerobic fermentation; **c** The NADH/NAD^+^ ratio in aerobic fermentation; **d** The NADPH/NADP^+^ ratio in aerobic fermentation; **e** The NADH/NAD^+^ ratio in microaerobic fermentation; **f** The NADPH/NADP^+^ ratio in microaerobic fermentation

As the glucose consumption of Sce-NOX was much faster, it can be expected that the NADH oxidase also increased the demand of NADH in anaerobic condition, since the glycolysis pathway is the main pathway to generate NADH. NADH homeostasis in response to an increase in NADH demand was achieved by the regulation of the glycolysis pathway, in accordance with a previous report of an NADPH oxidation system [17]. In our batch fermentation, the concentrations of the intracellular cofactors were measured at 26, 30, and 34 h. The intracellular NADH/NAD^+^ ratios were higher for Sce-NOX than for Sce-CON at all three time points, while the NADPH/NADP^+^ ratios were similar for both strains (Fig. 2). The NADH/NAD^+^ ratios were not consistent with previously reported results. Vemuri et al. reported that for carbon-limited as well as nitrogen-limited conditions, the NADH/NAD^+^ ratio was 20–50 % lower for the NOX strain than for the control strain [1]. To confirm our results, aerobic fermentation (500/100 mL Erlenmeyer flask sealed with eight-layer gauze; 32 °C; 200 rpm) and microaerobic fermentation (500/100 mL Erlenmeyer flask sealed with eight-layer gauze and one piece of kraft paper; 32 °C; 150 rpm) were conducted. The NADH/NAD^+^ and NADPH/NADP^+^ ratios in aerobic and microaerobic conditions showed the same trends as those in anaerobic conditions; the NADH/NAD^+^ ratios of Sce-NOX in the three oxygen supply models were higher than those of Sce-CON (Fig. 2c–f). These inconsistencies may be due to differences between the strains used in this and the other studies. First, the parental strains were different, which may have affected the engineered phenotypes. Second, the genomic backgrounds of the NADH oxidase genes used were different. In the current study, the gene from *L. lactis* was used, while the gene from *Streptococcus pneumoniae* was used in the study by Vemuri et al. [1]. Third, the fermentation conditions were different: complete medium was used in batch fermentation in our study, while aerobic fermentations were conducted in nitrogen-limited and carbon-limited chemostats by Vemuri et al. Altogether, these differences might have led to the different results.

Because the oxidation was increased by overexpression of the NADH oxidase, one would expect increased oxidation of NADH and the regeneration of NAD^+^, leading to a decreased NADH/NAD^+^ ratio. Thus, the increased NADH/NAD^+^ ratio in our study may seem paradoxical. A similarly paradoxical phenomenon has been reported for *S. cerevisiae* earlier; when aerobically growing cells of *S. cerevisiae* were shifted from glucose-limiting to glucose-rich conditions, the ATP level decreased by 40 % [18, 19]. As an ample amount of phosphate is available to the cells, one would expect a new steady state to occur, accompanied by an increased ATP/ADP ratio. This phenomenon was termed the “ATP paradox.” Many researchers have studied this phenomenon, using methods such as metabolic control analysis [20] and a core model consisting of a monocyclic interconvertible enzyme system [21]. Controlling the ATP concentration could be a subtle function of the relative activation of catabolic and anabolic routes. Similarly, the NADH/NAD^+^ ratio in our study might not be determined by the single action of NADH oxidase alone, as the heterologous enzyme most probably does not affect a specific metabolic reaction. Moreover, the much faster glucose consumption of Sce-NOX might provide a larger amount of NADH, and as expected, the glycerol synthesis pathway was not activated in the presence of ample NADH. These findings indicate that the bacterial NADH oxidase has a higher affinity toward NADH than the native NADH dehydrogenases, which has been suggested by Vemuri et al. on the basis of a genome-wide transcript analysis [1]. However, the exact reason of the higher NADH/NAD^+^ ratio observed for Sce-NOX remains unclear.

**Fig. 3:**
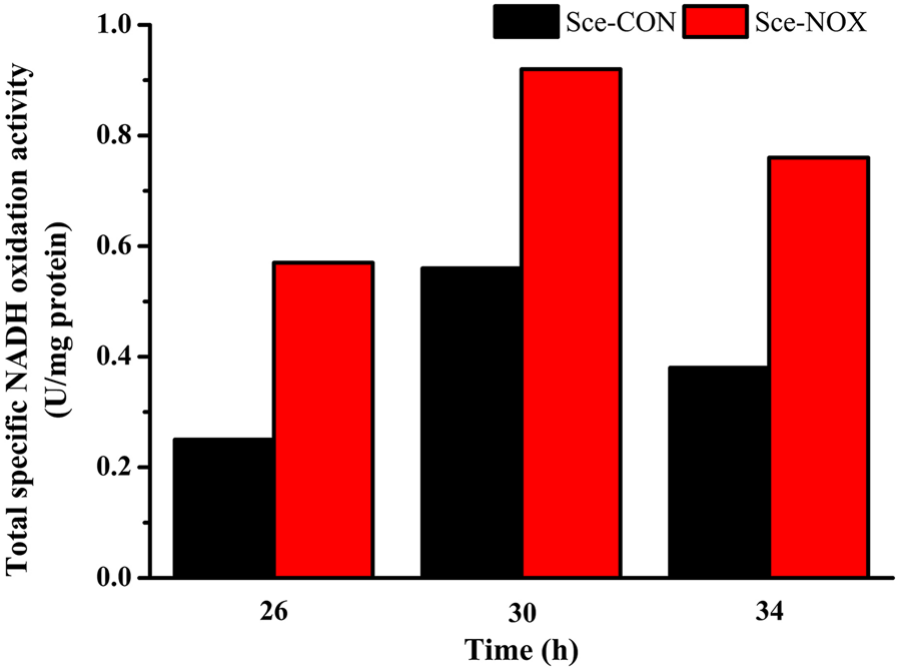
Total specific NADH oxidation activity in Sce-CON and Sce-NOX. In the batch fermentation, the total specific NADH oxidation activity of Sce-CON and Sce-NOX at 26, 30 and 34 h were measured

Next, the NADH oxidation capacity was measured. The assay for analyzing NADH oxidation is not specific for NADH oxidase and includes activity native to *S. cerevisiae* (e.g., NADH dehydrogenases) [1]. As shown in Fig. 3, Sce-NOX consistently exhibited greater NADH oxidation activity than Sce-CON at all three time points.

**Fig. 4:**
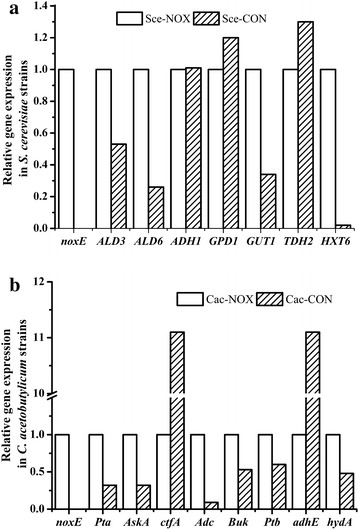
qRT-PCR analysis of *S. cerevisiae* and *C. acetobutylicum* strains. Cycle threshold (Ct) values from three technical replicates were averaged. Fold differences were calculated using the reference gene *ACT1* for *S. cerevisiae* strains the housekeeping gene CA_C0279 for *C. acetobutylicum* strains. **a** Results of qRT-PCR for genes related to ethanol and glycerol metabolism in *S. cerevisiae*. *noxE* NADH oxidase, *ALD3* aldehyde dehydrogenase, *ALD6* aldehyde dehydrogenase, *ADH1* alcohol dehydrogenase, *GPD1* glycerol-3-phosphate dehydrogenase, *GUT1* glycerol kinase, *TDH2* glyceraldehyde-3-phosphate dehydrogenase, *HXT6* hexose transporter. **b** Results of qRT-PCR for genes related in ABE and acid metabolism in *C. acetobutylicum*. *noxE* NADH oxidase, *Pta* phosphotransacetylase, *AskA* acetate kinase, *ctfA* butyrate-acetoacetate CoA-transferase, *Adc* acetoacetate decarboxylase, *Buk* butyrate kinase, *Ptb* phosphate butyryltransferase, *adhE* bifunctional acetaldehyde-CoA/alcohol dehydrogenase, *hydA* hydrogen dehydrogenase

qRT-PCR was conducted to assay the expression of *noxE* and genes related to ethanol and glycerol metabolism. As expected, *noxE* was not expressed in the control strain Sce-CON (Fig. 4a). On the other hand, it was effectively expressed in the recombinant strain Sce-NOX. The expression of *GUT1*, involved in the glycerol assimilation pathway, was upregulated, whereas the expression of *GPD1* gene, involved in the glycerol synthesis pathway, did not substantially change. The expression of *ALD6* was upregulated, suggesting that the conversion of acetaldehyde to acetate was stimulated, consistent with a previous report [1]. The detailed results and the calculative process were shown in the Additional file 1.

### Anaerobic fermentation by Cac-CON and Cac-NOX

Batch culture growth of the control strain Cac-CON and of the Cac-NOX strain overexpressing NADH oxidase in anaerobic condition was compared. After 72 h, the bubbles disappeared and the fermentation was completed. As shown in Table 3, when compared with Cac-CON, the metabolism of Cac-NOX changed obviously. The consumption of glucose and the concentrations of acetone, butanol, and ethanol (ABE) were all lower in Cac-NOX culture. For Cac-CON, the concentrations of ABE were 3.04, 10.43, and 6.24 g/L, respectively. For Cac-NOX, the concentrations of ABE were 2.17, 7.24, and 1.32 g/L, respectively, showing reductions of 28.62, 30.58, and 78.85 % as compared to Cac-CON. In contrast, the concentrations of acetic acid (1.17 ± 0.08 vs. 0.32 ± 0.02 g/L) and butyric acid (0.56 ± 0.01 vs. 0.11 ± 0.01 g/L) were more than five times higher for Cac-NOX. Different from the results obtained in *S. cerevisiae*, the intracellular NADH/NAD^+^ ratio of Cac-CON was 0.22 ± 0.02, while it was 0.15 ± 0.02 for Cac-NOX, i.e., a 31.82 % decrease. The deficiency of NAD(P)H hampered the conversion of acid into alcohol. A previous study reported that the addition of methyl viologen, which could shift the metabolism of *C. acetobutylicum* away from hydrogen production toward alcohol formation, could increase the intracellular NADH/NAD^+^ ratio [22]. However, when H_2_O_2_ was added into the medium, the intracellular NADH/NAD^+^ ratio was decreased, and the expression of genes encoding NADH-consuming enzymes in the central metabolism was repressed, increasing the availability of reducing power needed for reduction of H_2_O_2_ [22]. The addition of H_2_O_2_, similar to our operation, increased the loss of reducing power, which in turn decreased the intracellular NADH/NAD^+^ ratio and the production of alcohol. The NADH oxidation capacity in the *C. acetobutylicum* strains was measured at the end of fermentation. In Cac-CON, the NADH oxidation activity was below the detection limit, while an activity of 0.57 U/mg protein was noted for Cac-NOX. In our study, although the water-forming NADH oxidase did not improve the production of butanol as reported by Kawasaki et al. [5], it certainly did regulate the metabolism of *C. acetobutylicum*.

**Table 3:** Comparison of batch fermentation of Cac-CON and Cac-NOX

	Residual glucose (g/L)	Acetone (g/L)	Ethanol (g/L)	Butanol (g/L)	Acetoin (g/L)	Acetic acid (g/L)	Butyric acid (g/L)
Cac-CON	2.45 ± 0.12	3.04 ± 0.15	6.24 ± 0.31	10.43 ± 0.52	2.20 ± 0.11	0.32 ± 0.02	0.11 ± 0.01
Cac-NOX	26.82 ± 1.34	2.17 ± 0.11	1.32 ± 0.07	7.24 ± 0.36	0.59 ± 0.03	1.71 ± 0.08	0.56 ± 0.01

Each value is an average of three parallel replicates

qRT-PCR performed to assay the expression of *noxE* and genes related to ABE and acid metabolism revealed that *noxE* was not expressed in Cac-CON, while it was expressed in Cac-NOX (Fig. 4b). In accordance with the results of batch fermentation, the expression of *ctfA* and *adhE*, which are involved in the ABE synthetic pathway, was significantly downregulated. The expression of *Pta* and *AskA*, involved in acetic acid synthesis, was significantly upregulated. Finally, *hydA* expression was upregulated, indicating the increased loss of reducing power. The detailed results and the calculative process were shown in the Additional file 1.

Thus, overexpression of the water-forming NADH oxidase in the two different microorganisms, *S. cerevisiae* and *C. acetobutylicum*, could regulate the metabolism of both strains in anaerobic fermentation, confirming that the NADH oxidase has an alternative electron acceptor in anaerobic conditions, although the optimal and accepted electron acceptor of this enzyme is O_2_.

### Differential response of *S. cerevisiae* and *C. acetobutylicum* to overexpression of the water-forming NADH oxidase

**Fig. 5:**
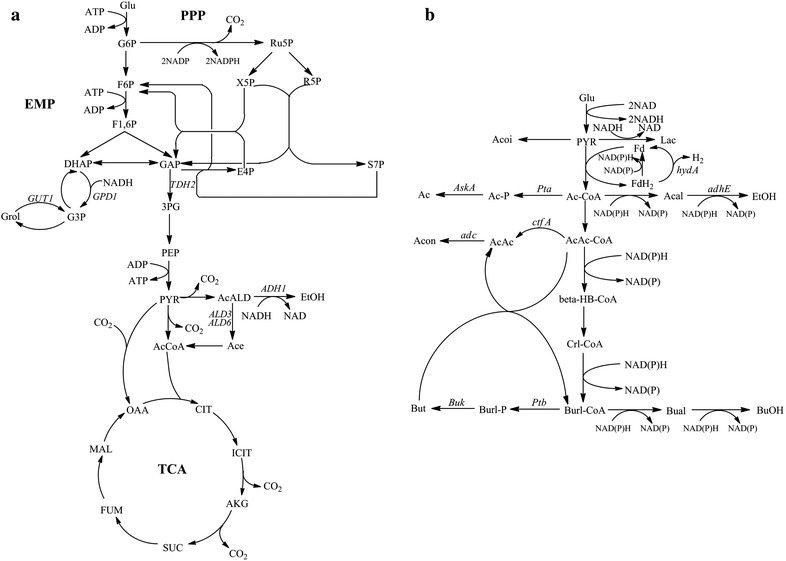
Metabolic networks of *S. cerevisiae* and *C. acetobutylicum*. **a** The metabolic network of *S. cerevisiae*; **b** The metabolic network of *C. acetobutylicum*

In *S. cerevisiae*, rapid consumption of glucose can lead to the accumulation of NADH; thus, lowering NADH accumulation by elevating either the rate of respiration or the direct oxidation of NADH is a logical approach to reduce overflow metabolism in *S. cerevisiae* (Fig. 5a). Glycerol is generated to reoxidize surplus cytosolic NADH that is formed in glycolysis [1]. Because of the outflow of intracellular acetaldehyde, glycerol branch shunting acts to reduce the efficiency of ATP regeneration. Shifting the end product of glucose metabolism from ethanol to glycerol abates ATP regeneration and increases ATP consumption. Therefore, the higher the glycerol accumulation, the lower the ATP regeneration efficiency is [23]. In this study, overexpression of the NADH oxidase elevated the rate of NADH oxidation in anaerobic condition. The elevated NAD^+^ regeneration increased the flux to glycolysis, which finally increased the production of ethanol.

In *C. acetobutylicum*, the decarboxylation and dehydrogenation of pyruvate are catalyzed by ferredoxin oxidoreductase. In addition to the generation of acetyl-CoA, this process involves the reduction of ferredoxin (Fd). The reduced ferredoxin FdH_2_ can transfer an electron to NAD(P)^+^ to generate NAD(P)H, which can be further used in the synthesis of ethanol and butanol. However, in the process of butanol fermentation, a large fraction of FdH_2_-derived hydrogen was not used to generate intracellular NAD(P)H but escaped in the form of hydrogen by the action of hydrogenase. Hydrogen escape led to a shortage of reducing power in the fermentation. It is the root cause of substantial accumulation of by-products, such as acetone and acetic acid, and the low yield of butanol [13]. Former studies have aimed at reducing the loss of reducing power to improve the butanol production capacity [24–26]; however, in our study, increasing the oxidation of NADH, which is equivalent to the decline of reducing power, most likely caused the accumulation of by-products and the lower production of the main products ABE. The imbalance of redox state decreased the glucose consumption and cell growth. As is well known, the fermentation of *C. acetobutylicum* is composed of two stages: production of acid during the exponential growth phase and production of alcohol during the stationary phase [8]. The low cell growth rate and the low intracellular concentration of NAD(P)H hampered transition to the alcohol production phase as the production of ethanol and butanol requires NAD(P)H (Fig. 5b).

### Survivability of Cac-NOX in different oxygen supply models

Multiple reports have mentioned that when exposed to oxygen, some anaerobic bacteria overexpress NADH oxidase to enhance their survival [5, 6, 27]. To investigate whether the water-forming NADH oxidase might act as an oxygen scavenger in *C. acetobutylicum*, we designed a series of experiments in different oxygen supply conditions using the same seed culture. The results showed that, regardless of shaking, both Cac-CON and Cac-NOX showed very poor cell growth in the bottles sealed with eight layers of gauze (and one piece of kraft paper). In these conditions, the cells could not form a membrane as under the normal conditions in 100-/50-mL screw-capped bottles. In addition, the consumption of glucose was quite slow, resulting in high concentrations of residual sugar (data not shown).

To determine the maximum level of oxygen supply that the *C. acetobutylicum* strains can tolerate, the effect of headspace on the growth of Cac-CON and Cac-NOX and production of solvent was studied (no nitrogen was sparged). The headspace volume fractions tested were 0, 25, 50 and 75 % [11]. As shown in Table 4, different headspace had little effect on Cac-CON. ABE, acetic acid, and butyric acid were produced at the same level in all four conditions. The production of ABE by Cac-NOX did not exceed that by Cac-CON in any of the headspace volumes tested, while the concentrations of acetic acid and butyric acid were higher. The highest concentration of butanol (8.79 ± 0.44 g/L) was obtained in the case of 75 % headspace. With decreasing headspace, the production of ABE declined while the production of acetic acid and butyric acid did not change obviously. We hypothesized above that the overexpression of the water-forming NADH oxidase in *C. acetobutylicum* would exacerbate the deficiency of reducing power, which would subsequently affect the production of ABE. However, the headspace experiments showed that upon overexpression of the NADH oxidase, higher concentrations of ABE could be obtained with larger headsspace in the screw-capped bottle. This finding was in accordance with the results reported by Al-Shorgani et al. [11].

**Table 4:** The influence of headspace to the control strain Cac-CON and the overexpressing NADH oxidase strain Cac-NOX

Products (g/L)	Headspace							
	Cac-CON				Cac-NOX			
	75 %	50 %	25 %	0 %	75 %	50 %	25 %	0 %
Acetone	5.12 ± 0.26	4.47 ± 0.22	5.08 ± 0.25	4.56 ± 0.23	4.90 ± 0.24	4.18 ± 0.21	2.99 ± 0.15	1.31 ± 0.07
Ethanol	2.07 ± 0.10	1.98 ± 0.10	2.11 ± 0.11	1.83 ± 0.09	0.77 ± 0.04	0.95 ± 0.05	0.48 ± 0.02	0.24 ± 0.01
Butanol	12.15 ± 0.61	11.96 ± 0.60	12.17 ± 0.61	11.78 ± 0.59	8.79 ± 0.44	8.37 ± 0.42	5.84 ± 0.29	3.29 ± 0.16
Acetic acid	0.84 ± 0.04	0.45 ± 0.02	0.52 ± 0.03	1.78 ± 0.09	1.56 ± 0.08	3.37 ± 0.17	1.91 ± 0.10	2.50 ± 0.12
Butyric acid	0.64 ± 0.03	0.28 ± 0.01	0.24 ± 0.01	0.32 ± 0.02	1.47 ± 0.07	0.32 ± 0.02	0.74 ± 0.04	0.45 ± 0.02

Each value is an average of three parallel replicates

### Alternative electron acceptors of the water-forming NADH oxidase in anaerobic condition

**Fig. 6:**
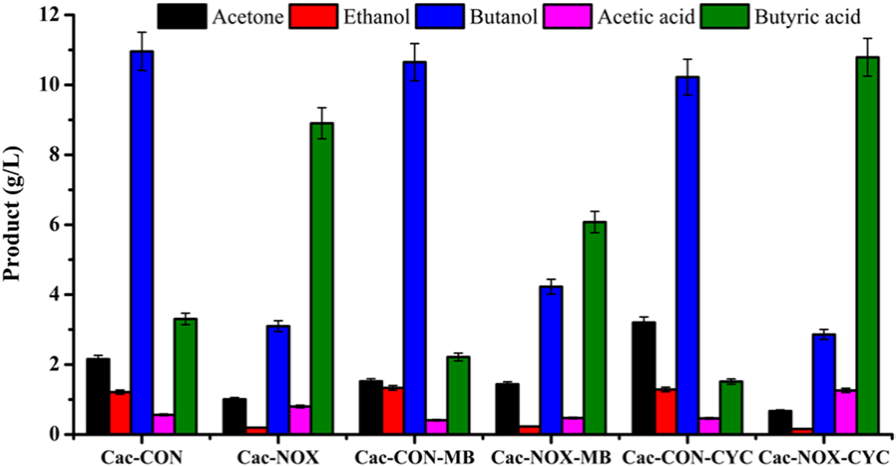
Influence of the potential electron acceptors on the metabolism of *C. acetobutylicum*. The potential electron acceptors methylene *blue* and cytochrome c were added into the medium through filter membranes before fermentation of Cac-CON and Cac-NOX

Our results indicated that the water-forming NADH oxidase has alternative electron acceptors in anaerobic condition. Currently, only limited reports focus on alternative electron acceptors. The H_2_O_2_-forming NADH oxidase has alternative electron acceptors, such as methylene blue, cytochrome c, *p*-nitroblue tetrazolium, 2,6-dichloroindophenol, and potassium ferricyanide in anaerobic conditions [7, 8]. Some of these were used in the *C. acetobutylicum* fermentation to assess whether they were the alternative electron acceptors in anaerobic conditions, as the difference between Cac-CON and Cac-NOX was more obvious than that between Sce-CON and Sce-NOX. Methylene blue, cytochrome c, *p*-nitroblue tetrazolium, and 2,6-dichloroindophenol were added into the fermentation medium through filter membranes at a concentration of 0.1 g/L. In the medium with *p*-nitroblue tetrazolium or 2,6-dichloroindophenol, both the Cac-CON and Cac-NOX strains could not grow at all, and all the products were nearly undetectable (data not shown). However, both Cac-CON and Cac-NOX could grow in the presence of methylene blue or cytochrome c. Methylene blue and cytochrome c had little influence on the metabolism of Cac-CON. The production of ethanol, butanol, and acetic acid remained at the same level. Cytochrome c led to a slight increase in the concentration of acetone (2.16 ± 0.11 vs. 3.20 ± 0.16 g/L) and a small decrease in that of butyric acid (3.30 ± 0.17 vs. 1.52 ± 0.08 g/L) (Fig. 6). In contrast, the production of metabolites in Cac-NOX culture changed a lot in the presence of the potential electron acceptors methylene blue or cytochrome c. In the case of methylene blue, the concentrations of ABE were all increased while those of acetic acid and butyric acid were decreased. The concentration of butanol produced by Cac-NOX increased from 3.10 ± 0.15 to 4.23 ± 0.21 g/L, and the concentration of butyric acid decreased from 8.90 ± 0.45 to 6.08 ± 0.30 g/L. In the case of cytochrome c, the production of ABE was slightly reduced, while that of acetic acid and butyric acid increased. The concentration of butyric acid increased from 8.90 ± 0.45 to 10.79 ± 0.54 g/L. These results showed that methylene blue could relieve the effects on the metabolic network of *C. acetobutylicum* strains, decline in reducing power, accumulation of by-products, and the lower production of the main products ABE, caused by the overexpression of the water-forming NADH oxidase, while cytochrome c aggravated the effects, possibly by exacerbating the imbalance of cofactors or the deficiency of reducing power. Methylene blue and/or the structural analogs could be the alternative elector acceptor of the water-forming NADH oxidase in anaerobic conditions.

## Conclusions

This study highlighted the role of overexpression of the water-forming NADH oxidase in *S. cerevisiae* and *C. acetobutylicum* in anaerobic conditions. In contrast to previous studies, which focused on the aerobic condition, the metabolism of the two different microorganisms could be regulated by this NADH oxidase in anaerobic fermentation, showing the potential usability of the recombinant *S. cerevisiae* strain in large-scale ethanol production. Larger headspace was better for the growth and ABE production of strain Cac-NOX in the screw-capped bottle. In addition, methylene blue and/or the structural analogs could be the alternative elector acceptor of the water-forming NADH oxidase in anaerobic conditions. However, the detailed mechanism underlying the higher NADH/NAD^+^ ratio in Sce-NOX than in Sce-CON could not be determined in this study and requires further research.

## Additional file

10.1186/s13068-016-0517-y Results of qRT-PCR of *S. cerevisiae* and *C. acetobutylicum.*

## Abbreviations

Sce-CON: *Saccharomyces cerevisiae* BY4741 containing the empty plasmid pYX212
Sce-NOX: *S. cerevisiae* BY4741 overexpressing the water-forming NADH oxidase encoded by *noxE*
Cac-428: *C. acetobutylicum* 428
Cac-CON: *C. acetobutylicum* 428 containing the empty plasmid pSY8
Cac-NOX: *C. acetobutylicum* 428 overexpressing the water-forming NADH oxidase encoded by *noxE*
ABE: acetone–butanol–ethanol
